# Is in-group bias culture-dependent? A meta-analysis across 18 societies

**DOI:** 10.1186/s40064-015-1663-6

**Published:** 2016-01-22

**Authors:** Ronald Fischer, Crysta Derham

**Affiliations:** Aarhus Institute of Advanced Studies, Aarhus, Denmark; Victoria University of Wellington, Wellington, New Zealand; Melbourne Clinical and Child Psychology Pty. Ltd., Albert Road Clinic, Melbourne, Australia

**Keywords:** Social identity theory, In-group bias, Uncertainty, Individualism-collectivism, Autonomy, Culture, Minimal group paradigm

## Abstract

We report a meta-analysis on the relationship between in-group bias and culture. Our focus is on whether broad macro-contextual variables influence the extent to which individuals favour their in-group. Data from 21,266 participants from 18 societies included in experimental and survey studies were available. Using Hofstede’s (1980) and Schwartz (2006) culture-level predictors in a 3-level mixed-effects meta-analysis, we found strong support for the uncertainty-reduction hypothesis. An interaction between Autonomy and real vs artificial groups suggested that in low autonomy contexts, individuals show greater in-group bias for real groups. Implications for social identity theory and intergroup conflict are outlined.

## Background

In-group bias (the tendency to favour one’s own group over other groups) has been one of the most well-supported psychological findings. The ground-breaking research by Tajfel et al. (1971) demonstrated that in-group bias can occur when group boundaries are completely meaningless, such as when using random letters or inconsequential artistic preferences. This phenomenon has been found to be so robust and stable, that researchers even started investigating potential genetic components (Lewis and Bates 2010). At the same time, both different experimental manipulations and the nature of manipulated groups may increase or decrease the in-group bias (for reviews, see for example Bettencourt et al. 2001; Brewer 1979; Tajfel 1982; Hewstone et al. 2002; Mullen et al. 1992). What has not been addressed previously is to what extent the larger macro-societal context may also have an influence on in-group bias. It is noteworthy that those structural variables which have been extensively discussed within the classic Social Identity Theory (SIT, Tajfel and Turner 1979, 1986), such as the nature of groups and their mutual relationships, are likely to vary systematically across different societies (Hofstede 1980). These more distal cultural variables have remained largely unexplored in research on in-group bias. Our question returns to the early writings by Tajfel who explicitly stated that group processes need to be understood within the context in which they are occurring and can not be assumed to be ‘universal’ (Tajfel et al. 1971, page 151).

In this study we focus on the effect of societal culture as one potential variable at a distal level affecting in-group bias both under minimal group conditions and in real-world contexts. Examining this question will allow us (a) to shed light on the boundary conditions of SIT, as well as (b) outline some potential mechanisms that drive in-group bias in both experimental and real-life situations. SIT is the dominant theory examining how individuals are linked to groups in intergroup contexts within societies. A different paradigm focusing on individual-group relationships has been individualism-collectivism, focusing on the salience of individual-group relations across societies (e.g., Smith and Bond 1998; Smith et al. 2013; Triandis 1995). A number of authors have noted cultural differences (Wetherell 1982; Yamagishi et al. 1998) and discussed whether social identity processes are more (or less) applicable in collectivist compared to individualistic societies (e.g., Brown et al. 1992; Hinkle and Brown 1990; Yamagishi et al. 1998). We address this issue directly. Regarding the second question, the mechanisms underlying social identity theory have been challenged (e.g., Hogg and Abrams 1990, 1988). A desire to increase self-esteem has been long argued to underlie intergroup discrimination (for a critical examination, see Aberson et al. 2000). A plausible alternative mechanism is uncertainty reduction (Hogg 2007). Previous experimental research has focused on task uncertainty, while recognizing that contextual uncertainty also may play a role. Here, we examine macro-contextual variables linked to uncertainty at the societal level. Therefore, cross-cultural work can help to examine plausible theoretical mechanisms that drive minimal group effects.

In summary, the aim of the study is to examine the strength of in-group bias in both experimental and real-word contexts and to link it to cultural differences in societal values. Therefore, we explore how distal macro-contextual (societal) effects, independent of specific structural intergroup variables (permeability, stability, legitimacy) within societies, influence overall in-group bias, which will allow for a greater contextualization and positioning of social identity in a globalized world.

## In-group bias

In-group bias is the positive evaluation of the in-group compared to the out-group (Brewer 1979). This evaluation can be accompanied by a preferential allocation of resources to in-group members, competition with out-group members and collaboration with in-group members or other perceptual, affective or behavioural effects that positively discriminate the in-group from the out-group, a condition that is often linked to ethnocentrism (Brewer 1979; Mullen et al. 1992). Tajfel and his students (Tajfel 1970; Tajfel et al. 1971) demonstrated that such in-group biases can be produced by a mere classification of individuals into random groups. This so-called ‘minimal group’ paradigm required that (a) individuals have no face-to-face contact, (b) group membership is completely anonymous, (c) there is no instrumental or rational link between the categorization and the experimental tasks to be completed by participants, and (d) the responses by participants have real implications for the groups, but not the responding individual (Tajfel et al. 1971, pp. 153–154). This experimental paradigm was an important element in the emergence of social identity theory (Tajfel and Turner 1979, 1986). Individuals are thought to strive for positive social identities to maintain positive self-esteem. This striving for self-enhancement is best achieved by intergroup social comparisons in which the in-group is positively differentiated from an out-group. The exact nature and process of social comparison depends on the relations between the groups and their perceived relative status, stability, legitimacy and permeability (Tajfel and Turner 1979). This direct link of the in-group bias to self-esteem motives has been debated and the current evidence is suggesting a more complex relationship (Aberson et al. 2000; Hogg and Abrams 1988).

Hogg (2000, 2007) developed uncertainty-identity theory as an extension of SIT. Hogg focused on the motivational processes underlying group identification and argued that self-uncertainty is a primary motive for identification with groups. To feel uncertain is an aversive psychological state and most people are motivated to reduce this sense of uncertainty. Put simply, by identifying with a social group, people derive a sense of meaning and belonging. This subjective group membership in turn provides prescriptive norms and guidelines for appropriate behaviour (“this is what we do”) that help individuals to navigate an uncertain world. The uncertainty reduction mechanism has been supported in a number of experiments, mainly focusing on task uncertainty (Grieve and Hogg 1999; Mullin and Hogg 1999).

Despite debate around the relevant process mechanism leading to in-group bias, the relative effect of in-group bias itself was found to be highly stable and robust. A number of meta-analyses have summarized the empirical research to date and have demonstrated that the effect is replicable and consistent (Bettencourt et al. 2001; Buhl 1999; Mullen et al. 1992) and it has been accepted as an important theoretical process in explaining intergroup relations (Hewstone et al. 2002).

## Uncertainty avoidance and in-group bias

As discussed above, uncertainty has been suggested to play a major role in explaining intergroup discrimination. Hogg (2000, 2007) distinguished two types of sources of uncertainty: uncertainty arising from the immediate task and uncertainty inherent in the larger social context. Larger context variation in uncertainty has not received much attention in the social identity literature, but there is systematic variability in the management of uncertainty across modern societies. Hofstede’s (1980) seminal study of culture defined uncertainty avoidance as the extent to which individuals within societies are socialized to avoid uncertain situations by establishing formal rules and structures. Higher uncertainty avoidance is a situational concern with reducing uncertainty, which can be predicted to increase in-group bias. We therefore test to what extent this society level concern with avoiding uncertainty is related to in-group bias. Greater uncertainty avoidance should be associated with greater in-group bias (Hypothesis 1).

### Individualism-collectivism and in-group bias

Individualism-collectivism (Hofstede 1980; Triandis 1995) has emerged as a major dimension in cultural research that differentiates contexts in which individuals are thought to be self-reliant, autonomous, independent, driven by their own attitudes, beliefs and convictions and endorsing a rational attitude towards group membership, with cultural contexts that socialize individuals to be interdependent, to rely on one’s duties, norms and morals dictated by the group in deciding on actions and to form strong emotional and affective bonds with groups. The former is more typical of Western Europe and North American societies, where the latter is a typical socialization pattern in much of Asia, Africa, Eastern and Southern Europe and South America.

Associations between individualism-collectivism and in-group bias in both directions have been observed, potentially driven by different mechanisms. First, collectivism might be associated with higher in-group bias. Hinkle and Brown (1990) suggested that social identity processes are more likely to take place in collectivistic kind of groups, because collectivistic groups or individuals are more likely to worry about in-group identification. A slightly different argument was provided by Hogg (2007). As discussed above, uncertainty reduction is one potential driver for intergroup discrimination. Therefore, greater uncertainty avoidance should lead to stronger in-group association, which in turn leads to an empirical merging of uncertainty avoidant and collectivist behavioural pattern. As a consequence, empirically it will be difficult to separate in-group attachment and uncertainty mechanisms because the two dimensions are intrinsically linked. In line with these arguments, uncertainty avoidance and collectivism are highly correlated in Hofstede’s data set (see Hogg 2007) or did not emerge as separate dimensions in other cultural frameworks such as Schwartz’ (1994). These arguments are also consistent with the strategic equilibrium dilemma noted by Yamagishi et al. (1998), who predicted that in closed-knit groups, people practice in-group favouritism because it gives them a competitive advantage and reduces uncertainty.

In contrast to these positive associations of in-group bias with collectivism, opposing relationships may also be plausible. Hogg (2000, 2007) observed that stable identities in a pre-modern society have been replaced by an atomistic individual-oriented society, which is accompanied by an erosion of traditional community links and stable collective identification targets. This lose connection of the self to various possible groups then leads to a greater concern with group identities as well as a heightened need to establish and maintain links to in-groups. In more collectivistic settings, people do not need to worry about their identities or level of inclusion in the group since individual-group links are stable and not negotiable. People have typically little choice to stay or leave a group and fewer in-groups pervade more aspects of everyday life compared to more individualistic societies, where multiple potential in-groups are available, but temporally and geographically separated. This so-called ‘postmodern paradox’ therefore leads to individuals in highly individualistic societies to yearn for collective affiliations. The evidence supporting either of the two mechanisms is mixed (e.g., Brown et al. 1992; Capozza et al. 2000).

However, these two hypotheses may not be conflicting with each other. Yamagishi et al. (1998) suggested that collectivism is more strongly related to in-group favouritism because it provides in-groups with a strategic advantage (see also Hinkle and Brown 1990). In contrast, Hogg (2000) speculated that the ‘postmodern paradox’ increased the need of citizens in individualistic societies to identify with various possible in-groups. The minimal group paradigm uses arbitrary group distinctions, to the extent that people in individualistic societies ‘crave’ identification, we could expect a stronger bias towards these arbitrary groups. In contrast, in collectivist societies with more stable group membership there is less need for people to identify with novel groups, but in contrast it is identification with real groups that is most important for collectivists. Individuals have been socialized to feel emotionally attached and strongly connected to their in-groups, hence, in-group bias could be expected to be larger for real groups compared to randomly created groups in experimental settings that are likely to have little affective value for people in collectivist settings. This integration suggests an interaction hypothesis (Hypothesis 2): in-group bias is strongest in collectivistic contexts when focusing on real-groups and weakest in collectivistic settings when focusing on artificial groups. In-group might be intermediate in individualistic contexts with both real and artificial groups.

### Societal value dimensions

We have identified two major dimensions along which societies differ that might be related to in-group bias. The societal variables of interest are related to individual-group relations (e.g., individualism-collectivism), and the extent to which uncertainty is tolerated or not (e.g., uncertainty avoidance). We examine the effect of variables derived from two different value frameworks (Hofstede 1980; Schwartz 1994, 2006). These dimensions were derived from large scale studies in which individuals answered batteries of questions about their behaviours, attitudes, beliefs and values. The corresponding scores therefore reflect modal tendencies of attitudes, beliefs and behaviours of groups of individuals within a society. Individuals in all societies have options concerning their beliefs and behaviours and can engage in particular behaviours if the context allows it. Societal level dimensions of culture indicate that certain attitudes, beliefs or behaviours are more frequent in some societies compared to other societies, therefore reflecting overall behavioural tendencies of groups of individuals within a society.

In this study, we ask to what extent such tendencies are related to in-group bias. The classic cultural framework was developed by Hofstede (1980, 2001). He sampled employees in IBM and originally described four major dimensions. The dimensions of interest for us are individualism-collectivism and uncertainty avoidance. This framework has been extensively studied and is the most widely used dimensional framework of societal culture (Taras et al. 2010).

Schwartz (1994) developed an alternative framework that shows some overlap, but also deviates in important aspects. Based on ratings from teachers and students, he distinguished autonomy (subdivided into intellectual and affective components) from embeddedness (originally conservatism). This dimension is similar to individualism-collectivism, but emphasises more being embedded in social groups and deriving meaning and identity from belonging to inclusive and strong in-groups compared to the opposing end where individuals are seen as autonomous in their thoughts and actions and are freer to act in line with their self-interests. Schwartz (1994) argues that his autonomy versus embeddedness dimension is closer to the original ideal of individualism-collectivism (e.g., Triandis, 1995). Having separate dimensions for the opposing ends of this assumed underlying dimension (see Fischer et al. 2009) allows us to separate the differential effects of individualism (autonomy) versus collectivism (embeddedness).

Schwartz (1994) did not identify a separate societal dimension of uncertainty avoidance. However, the notions of security values (Schwartz 1992) which are a central element of embeddedness values reflect a concern with avoiding insecurity and risks, and maintaining stability and order in one’s surroundings. As noted above, uncertainty and collectivism are potentially strongly intertwined and might be difficult to separate empirically (see Smith et al. 2006).

In summary, Hofstede and Schwartz provide alternative frameworks for measuring individualism-collectivism. Only Hofstede provides separate scores for uncertainty avoidance. For the sake of consistency, we continue using the Hofstede labels, unless specifically referring to Schwartz’ dimensions.

### The current study

In this study we explore societal effects on in-group bias. To derive a measure of in-group bias, we conducted a meta-analysis of experimental and correlational studies that reported an index of in-group bias. We were not interested in more subtle variations in the effect (e.g., Bettencourt et al. 2001), but were interested in whether the overall level of in-group bias across conditions or group targets is influenced by more distal macro-contextual variables, independent of the structural relations between the groups within a society. Given the non-specific and broad nature of culture-level dimensions, by not paying attention to finer experimental distinctions or subtleties our approach provides a more conservative estimate of the influence of societal context on this psychological phenomenon. This is also based on pragmatic reasons as it allows us to pool a larger set of studies by focusing on more inclusive criteria. Unfortunately, to date there are not enough studies available across all the societies included in our study that would allow finer coding of structural variables within each society.

## Methods

### Literature search and effect size coding

We conducted a PsycINFO and Web of Science electronic data base search in November 2009 using the key words ‘minimal group’ and ‘ingroup/in-group bias’. We also consulted the reference lists of published meta-analyses on in-group bias and minimal group studies (Aberson et al. 2000; Mullen et al. 1992, Robbins and Krueger 2005). A total of 498 articles with these key words were found. In addition to these published articles, a total of 20 unpublished articles or doctoral theses were directly obtained from authors via email (to the best of our knowledge these studies are still unpublished at the time of writing). The search strategy was focused on maximizing studies that were not conducted in the UK or USA (as most research had been conducted there).

The inclusion criteria for the studies were the following. First, the study needed to have been conducted using a version of the minimal group paradigm or have included some measure of in-group bias (rating, recognition or allocation tasks). Second, the national background of participants should have been stated explicitly. We did not include migrant samples (e.g., Polynesians in NZ, Wetherell 1982) in our study, but minorities that were resident in a country were included. Third, sufficient information to compute an effect size for in-group bias should have been available.

We coded additional parameters. We are interested in overall in-group bias, yet status differences are the most consistent predictor of variability in in-group bias (Bettencourt et al. 2001; Buhl 1999; Mullen et al. 1992). If possible, we coded one overall estimate of in-group bias across all other conditions. If not, we coded status of the groups (−1 lower status, 0 equal status, 1 higher status) in line with Mullen et al. (1992) and Buhl (1999). Overall in-group bias collapsed across status was coded as equal status. If other conditions were included that were intended to experimentally decrease or eliminate in-group bias (e.g., uncertainty reduction, Grieve and Hogg 1999), we did not include those conditions in our coding. If there were multiple dependent variables, we averaged them per sample. In essence, our coding focused on overall estimates of in-group bias, averaging over variations in status, group size and type of dependent variable. If authors reported both in-group bias and out-group derogation, only in-group bias was coded. There was not sufficient information across studies conducted in the various societies to consistently differentiate in-group favouritism from out-group derogation (e.g., Bettencourt et al. 2001) or any finer differentiation of socio-structural variables as discussed in SIT research.

In-group bias was coded in the direction that greater emphasis was given to an in-group (vs an out-group). Therefore, a positive effect size shows a bias towards the in-group in ratings, allocations of points or money or recognition of group members. A negative sign indicates a bias towards the out-group. All effect sizes were converted to correlation coefficients (Rosenthal 1991) and then r-to-z transformed. The inverse variance weight was based on the sample size (n − 3).

The final data set contained 269 samples from 121 articles based on a total N of 21,266 participants from 18 societies. See Table 1 for the average in-group bias by society together with standard errors, confidence intervals and number of samples. The list of studies and coded information is available from the first author.

**Table 1:** Mean in-group bias per country, standard errors, 95 % confidence intervals and number of samples

	Mean in-group bias	Standard error	−95 % confidence interval	+95 % confidence interval	Number of samples
Australia	0.51	0.11	0.29	0.73	7
Belgium	0.57	0.18	0.22	0.92	3
Canada	0.24	0.09	0.05	0.42	10
Finland	0.66	0.17	0.33	0.98	3
Germany	0.37	0.05	0.27	0.47	45
Israel	0.31	0.13	0.05	0.58	5
Italy	0.61	0.11	0.38	0.83	7
Japan	0.31	0.27	−0.23	0.84	2
South Korea	0.45	0.15	0.14	0.75	4
Mexico	0.28	0.19	−0.09	0.64	3
Netherlands	0.39	0.09	0.21	0.56	11
New Zealand	0.45	0.10	0.25	0.65	8
Portugal	0.99	0.25	0.51	1.48	2
South Africa	0.27	0.22	−0.16	0.70	2
Spain	0.55	0.31	−0.06	1.15	1
Sweden	0.22	0.21	−0.19	0.62	2
UK	0.23	0.04	0.16	0.30	80
USA	0.46	0.04	0.39	0.53	74

### Country level indicators

We used country-means for uncertainty avoidance and individualism reported by Hofstede (1980, 2001). We used the averaged teacher and student scores for autonomy versus embeddedness from Schwartz (1994). Data for all 18 countries were available. The validity of these indicators is extensively discussed in Hofstede (2001) and Schwartz (1994, 2006).

### Meta-analytical strategy

Meta-analysis is a set of techniques that statistically combines the results of two or more independent studies to provide an overall answer to a question of interest (Everitt and Wykes 1999). In our analysis we are using a multi-level mixed effect model. Most meta-analyses use a fixed effects model (Field 2003; Lipsey and Wilson 2001). Effect sizes are seen as direct replications of each other and it is assumed that samples come from the same population (only subject-level sampling error is estimated). Although convenient, this assumption is not justified in most cases (Field 2003). A random model in contrast presupposes that studies are randomly drawn from a larger population of studies. Therefore, both subject-level sampling error and variability between samples are considered (van den Noortgate and Onghena 2001; Lipsey and Wilson 2001). Random effects models provide more adequate representations of most meta-analytical data sets (Konstantopoulos and Hedges 2004). Mixed effect models use a combination of both approaches, as they estimate both subject-level and study-level variation in effect sizes, but then allow testing of whether study variability is systematic and explicable by specific context variables beyond random variation (see further discussions in Field 2003; Hox and de Leeuw 2003; Konstantopoulos and Hedges 2004; van den Noortgate and Onghena 2001).

A significant advantage of mixed effects models is that findings can be generalized beyond the specific samples included in the meta-analysis. The assumption is that studies are assumed to be random samples from a larger population of studies. Therefore, the results can be generalized to other studies not yet conducted or to studies that were not included in the meta-analyses. Taking into account sampling of studies makes the results more conservative.

#### Multilevel approach

Since we have studies nested in countries, this nesting needs to be considered. Therefore, we developed a 3-level structure to account for dependencies with countries, where effect sizes are level 1, study characteristics are level 2 and country is level 3 (see Fischer and Mansell 2009 for a discussion of this approach). This set-up also allows us to control for a number of potentially important confounding variables. We first entered the publication year, status of groups and whether groups were artificial or real. We then controlled for any significant effect before entering the variables of interest at level 3 (society level). The effects of interest are (a) the effects of society level variables at level 3 on the intercepts at level 1 (main effects of context variables) and (b) the effects of country level variables at level 3 on the effects of level 2 (study characteristics) on the intercepts at level 1 (interaction between group type with country-level characteristics on in-group bias). Given the relatively small number of samples, we entered each predictor variable individually and then ran a final robustness test where all significant predictors were entered together.

## Results

The overall in-group bias using a random effects method in our sample was 0.369, standard error 0.021, with the 95 % confidence intervals stretching from 0.327 to 0.410. This bias was highly significant: z = 17.37, p < 0.0001. There was significant variation in effect sizes: Q (268) = 2224.06, p < 0.0001. Societal differences accounted for 8.75 % of the variability. This is comparable to other cultural differences in psychological variables (Fischer and Schwartz 2011).

We first estimated the effects of sample characteristics at level 2. Entering the publication year as a group-mean centred variable and both group status and group type as unstandardized predictors, the effects for year (γ = 0.003, t_robust_ = 0.99, p = 0.34) and group type (γ = 0.080, t_robust_ = 1.43, p = 0.17) were not significant. The effect for group status was significant (γ = 0.051, t_robust_ = 3.46, p = 0.003). Higher status groups show higher in-group bias. Removing year from the equation, status remained significant (γ = 0.050, t_robust_ = 4.54, p = 0.001) and type became marginally significant (γ = 0.096, t_robust_ = 1.92, p = 0.07). Real groups showed higher in-group bias. The two study variables together accounted for about 71.39 % of the variance in in-group bias across societies. The variance component for group type was marginally significant (χ^2^ [5] = 9.66, p = 0.08), but there was no significant variability in the effect for status: χ^2^ [5] = 1.28, p > 0.50.

### Hofstede

Supporting hypothesis 1, greater uncertainty avoidance was associated with more in-group bias: γ = 0.004, t_robust_ = 3.12, p = 0.007. The interaction with real vs artificial groups was not significant: γ = 0.001, t_robust_ = 0.00, p > 0.50. Uncertainty avoidance explained 79.02 % of the remaining variance between societies, after accounting for study effects at level 2.

Testing the effect of individualism, the main effect was not significant: γ = −0.001, t_robust_ = −1.38, p = 0.19. Hypothesis 2 was not supported, because the interaction effect between individualism and group type (real vs artificial) was not significant (but in the predicted direction): γ = −0.004, t_robust_ = −0.99, p = 0.34.

### Schwartz values

**Fig. 1:**
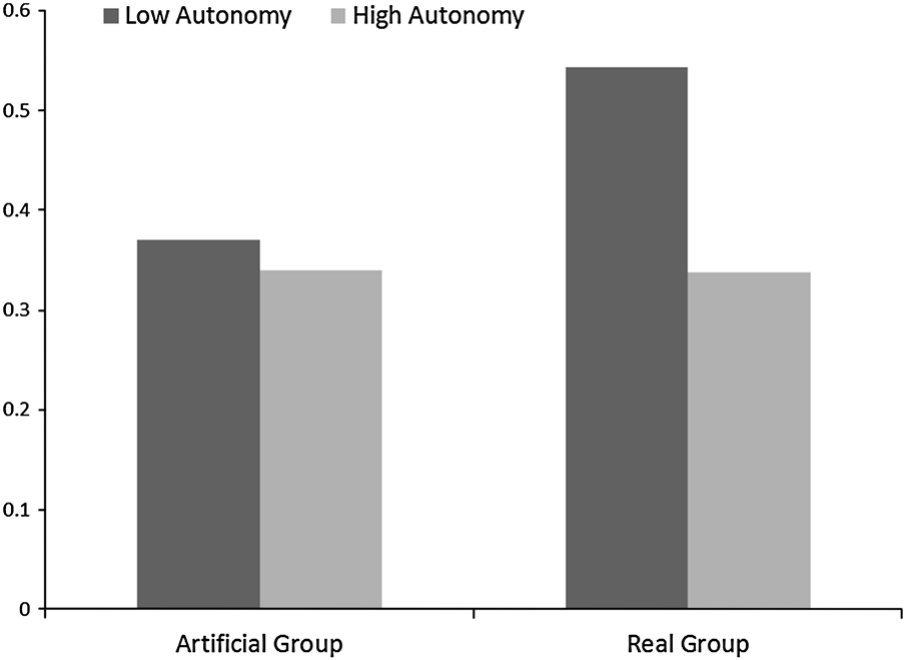
Interaction between autonomy and group target

Examining the effects of autonomy, the main effect was not significant: γ = −0.054, t_robust_ = −0.69, p > 0.50. Hypothesis 2 was supported because the interaction between group type and autonomy was significant: γ = −0.45, t_robust_ = −3.34, p = 0.004. Figure 1 shows the relationship. In line with hypothesis 2, in-group bias is stronger for real groups in less autonomous contexts compared to more autonomous contexts. Autonomy explained 87.95 % of the variability in differential effect of group type on in-group across societies.

Examining the effect of embeddedness, the main effect was not significant: γ = −0.019, t_robust_ = −0.32, p > 0.50. The interaction was not significant: γ = 0.262, t_robust_ = 1.50, p = 0.15. Although hypothesis 2 was not supported, the direction of the effect was in the expected direction and explained 45.65 % of the variability across societies.

### Robustness analysis

To test the robustness of all the findings, we entered all the significant effects in a single model (uncertainty avoidance, autonomy on in-group bias, interactions between group type and autonomy). Uncertainty avoidance remained significant: γ = 0.004, t_robust_ = 2.98, p = 0.009. Autonomy was not significant as before: γ = −0.053, t_robust_ = −0.083, p = 0.42. The interaction between type of group and autonomy remained significant: γ = −0.515, t_robust_ = −4.05, p = 0.001, but the interaction with uncertainty was not significant (as before): γ = 0.001, t_robust_ = 0.32, p > 0.50.

## Discussion

We examined in-group bias across 18 societies. The overall effect was significant and of similar moderate magnitude as in previous meta-analyses (e.g., Bettencourt et al. 2001; Mullen et al. 1992). This suggests that in-group bias is a relatively universal phenomenon. At the same time, the magnitude of the effect varied systematically across societies. In more uncertainty avoidant contexts, in-group bias increased. This finding supports uncertainty-avoidance theory (Hogg 2000). Identification with groups allows for reduction in uncertainty through allowing individuals to find a sense of who they are, and what to expect of those in and outside of one’s group (but see Fischer 2013).

Our analysis was also able to shed some light on the previously encountered inconsistent results in relation to individualism-collectivism related dimensions. Individualism-Collectivism is related to in-group bias only under specific conditions. It appears that individuals in less autonomous (less individualistic and more collectivistic) societies exhibit more in-group bias if the targets are real-life groups. In contexts where individuals are not strongly embedded in naturally occurring groups, a longing for identification may lead to greater bias favouring novel groups. However, in contexts where individuals are strongly embedded in their groups, in-group bias may become stronger if the focus is on those groups with which individuals identify. Therefore, the stronger identification with their in-groups will be expressed in in-group bias for those groups they feel strongly identified with, especially if the intergroup context is made salient and refers to relevant groups (Brown 2000).

Our results have a number of theoretical and practical implications. First, the findings suggest that intergroup conditions have a significant likelihood to deteriorate in high uncertainty salient contexts. Although plausible, this issue has not received much attention. To date, theoretical and empirical work has emphasized the effects of threat (Stephan and Stephan 2000). The closely related affective state of uncertainty is likely to show similar effects on intergroup relations. Our results certainly demonstrate that a societal concern with uncertainty avoidance increase the likelihood to favour the in-group over the out-group. These biases can have significant real-world implications if we convert it into money, jobs, access to basic resources or health, education, and social services. The overall levels of uncertainty and the general social climate can potentially have a large impact on intergroup relations. For example, increases in unemployment as one potential antecedent of experienced uncertainty has been shown to be associated with increased prejudice against Muslims in Europe (Strabac and Listhaug 2008). Cohrs and Stelzl (2010) found that when levels of unemployment increased, dominance beliefs were more strongly related to anti-immigrant attitudes. These findings suggest that additional to specific structural variables of the groups as typically studied in SIT research (legitimacy, permeability, stability), there are larger macro-contextual processes that influence all groups and potentially acerbate intergroup processes. We could not directly test such interaction effects, but it is plausible that general society-wide processes such as levels of uncertainty (or individual-group relations or hierarchy beliefs) have an impact on the specific structural variables tested in SIT research. Therefore, the interactions between legitimacy, permeability and group status may be weakened or strengthened depending on the larger societal context.

Second, we demonstrated one instance of how general psychological theories can be tested and extended through cross-cultural analyses. Treating the world as a natural laboratory, researchers can identify macro-level variables with systematic variation around the world that are theoretically meaningful. Using these variables, effects identified in rigorous laboratory experiments with high internal validity can be tested for their external validity. Such naturalistic quasi-experimental studies can also highlight areas for further research if some unexpected patterns emerge.

Third, the findings suggest that broad contextual variables influence responses of individuals in specific experimental settings. One question that arises is whether individuals are higher in uncertainty avoidance themselves or whether the general social climate has an effect. Hogg (2000, 2007) speculated that individual differences may have some facilitating influence. Experimental work (e.g., Grieve and Hogg 1999) has focused on task uncertainty. It would be interesting to tease apart the individual difference (socialization) component from both task demands and the overall social climate. One option would be to cross task demands and generalized uncertainty (e.g., through priming; see Van den Bos 2001) to examine the unique impact of either variable on identification processes.

### Limitations

We reported a meta-analysis of in-group bias. One limitation is that the available information was scarce. Often researchers did not report sufficient information to allow calculation of overall in-group effect sizes. Contacting authors to obtain information was not a very successful strategy since we only received 5 responses with only 2 of these being able to provide the information needed. Authors should be more conscientious about reporting experimental details and results.

Because of this lack of information, we averaged across experimental conditions and dependent variables. This results in a rather crude and unspecific measure of in-group bias. Our measure is similar in overall magnitude to previous meta-analyses, so this is re-assuring. However, we might have missed subtle effects (interactions between study characteristics and country-level characteristics). As desirable as this may be, the information from studies available from these studies did not allow finer coding without losing too much information. Specifically, a separation of in-group bias into in-group favouritism and out-group derogation would be informative. However, too few studies have used designs that allow the separation of these effects.

The inclusion of SIT variables such as permeability, stability and legitimacy are also desirable. It can be speculated that society-level processes interact with these structural group-level variables and differentially affect in-group bias. This is an important avenue for further research. In short, we had to make trade-offs between maximizing samples from as many countries as possible while not coding all experimental characteristics or paying closer attention to experimental designs and discarding much information from less often studied contexts. The fact that we found significant results in line with theoretical predictions is evidence of the robustness of the overall effects. Future meta-analysis with more primary studies could examine some of these interesting experimental variations.

## Conclusion

We reported a meta-analysis on in-group bias across 18 societies. One of our overall strengths was using a 3-level mixed effects meta-analysis procedure. This allows us to generalize our findings to other studies not included in our data base. We found a robust and consistent effect of uncertainty avoidance in line with uncertainty-identity theory. Greater concern at the societal level with uncertainty was associated with greater in-group bias. This has significant implications for the understanding and intervention in intergroup conflicts. Conflicts are more likely to escalate in contexts where uncertainty is salient. One potential intervention strategy could be to address general levels of uncertainty in a society.
